# Author Correction: Rapid increase in the risk of heat-related mortality

**DOI:** 10.1038/s41467-023-44107-z

**Published:** 2024-09-16

**Authors:** Samuel Lüthi, Christopher Fairless, Erich M. Fischer, Noah Scovronick, Ben Armstrong, Micheline De Sousa Zanotti Stagliorio Coelho, Yue Leon Guo, Yuming Guo, Yasushi Honda, Veronika Huber, Jan Kyselý, Eric Lavigne, Dominic Royé, Niilo Ryti, Susana Silva, Aleš Urban, Multi-Country Multi-City (MCC) collaborative research network, Ben Armstrong, Niilo Ryti, Antonio Gasparrini, Rosana Abrutzky, Yuming Guo, Shilu Tong, Micheline de Sousa Zanotti Stagliorio Coelho, Paulo Hilario Nascimento Saldiva, Eric Lavigne, Patricia Matus Correa, Nicolás Valdés Ortega, Haidong Kan, Samuel Osorio, Dominic Roye, Souzana Achilleos, Jan Kyselý, Ale Urban, Hans Orru, Ene Indermitte, Marek Maasikmets, Jouni J. K. Jaakkola, Mathilde Pascal, Alexandra Schneider, Veronika Huber, Susanne Breitner, Klea Katsouyanni, Antonis Analitis, Evangelia Samoli, Hanne Krage Carlsen, Fatemeh Mayvaneh, Hematollah Roradeh, Patrick Goodman, Ariana Zeka, Raanan Raz, Paola Michelozzi, Francesca de’Donato, Matteo Scortichini, Massimo Stafoggia, Masahiro Hashizume, Yoonhee Kim, Chris Fook Sheng Ng, Yasushi Honda, Barrak Alahmad, John Paul Cauchy, Magali Hurtado Diaz, César De la Cruz Valencia, Ala Overcenco, Danny Houthuijs, Caroline Ameling, Shilpa Rao, Gabriel Carrasco, Xerxes Seposo, Paul Lester Carlos Chua, Susana das Neves Pereira da Silva, Joana Madueira, Iulian Horia Holobaca, Simona Fratianni, Ivana Cvijanovic, Malcolm Mistry, Noah Scovronick, Fiorella Acquaotta, Rebecca M. Garland, Ho Kim, Whanhee Lee, Aurelio Tobias, Carmen Íñiguez, Bertil Forsberg, Ana Maria Vicedo-Cabrera, Martina S. Ragettli, Yue Leon Guo, Shih-Chun Pan, Shanshan Li, Francesco Sera, Pierre Masselot, Valentina Colistro, Michelle Bell, Antonella Zanobetti, Joel Schwartz, Tran Ngoc Dang, Do Van Dung, Antonio Gasparrini, David N. Bresch, Ana M. Vicedo-Cabrera

**Affiliations:** 1 Institute for Environmental Decisions, ETH Zurich, Zurich, Switzerland; 2 Federal Office of Meteorology and Climatology MeteoSwiss, Zurich, Switzerland; 3 Institute for Atmospheric and Climate Science, ETH Zurich, Zurich, Switzerland; 4 Gangarosa Department of Environmental Health. Rollins School of Public Health, Emory University, Atlanta, GA USA; 5 Department of Public Health Environments and Society, London School of Hygiene & Tropical Medicine, London, UK; 6 Department of Pathology, Faculty of Medicine, University of São Paulo, São Paulo, Brazil; 7 Environmental and Occupational Medicine, National Taiwan University (NTU) College ofMedicine and NTU Hospital, Taipei, Taiwan; 8 National Institute of Environmental Health Science, National Health Research Institutes, Zhunan, Taiwan; 9 Graduate Institute of Environmental and Occupational Health Sciences, NTU College of Public Health, Taipei, Taiwan; 10 Climate, AirQuality Research Unit, School of PublicHealth and PreventiveMedicine, Monash University, Melbourne, Australia; 11 Center forClimateChangeAdaptation, National Institute for Environmental Studies, Tsukuba, Japan; 12 IBE-Chair of Epidemiology, LMU Munich, Munich, Germany; 13 Department of Physical, Chemical and Natural Systems, Universidad Pablo de Olavide, Sevilla, Spain; 14 Institute of Atmospheric Physics, Czech Academy of Sciences, Prague, Czech Republic; 15 Faculty of Environmental Sciences, Czech University of Life Sciences, Prague, Czech Republic; 16 School of Epidemiology & Public Health, Faculty of Medicine, University of Ottawa, Ottawa, ON Canada; 17 Environmental Health Science and Research Bureau, Health Canada, Ottawa, ON Canada; 18 CIBER of Epidemiology and Public Health, Madrid, Spain; 19 Climate Research Foundation (FIC), Madrid, Spain; 20 Center for Environmental and Respiratory Health Research (CERH), University of Oulu, Oulu, Finland; 21 Department of Epidemiology, Instituto Nacional de Saúde Dr. Ricardo Jorge, Lisbon, Portugal; 22 Centre for Statistical Methodology, London School of Hygiene & Tropical Medicine, London, UK; 23 Centre on Climate Change & Planetary Health, London School of Hygiene & Tropical Medicine, London, UK; 24 Institute of Social and Preventive Medicine, University of Bern, Bern, Switzerland; 25 Oeschger Center for Climate Change Research, University of Bern, Bern, Switzerland; 26 Environment & Health Modelling (EHM) Lab, Department of Public Health Environments and Society, London School of Hygiene & Tropical Medicine, London, United Kingdom; 27 Universidad de Buenos Aires, Facultad de Ciencias Sociales, Instituto de Investigaciones Gino Germani, Buenos Aires, Argentina; 28 Department of Epidemiology and Preventive Medicine, School of Public Health and Preventive Medicine, Monash University, Melbourne, Australia; 29 School of Public Health and Social Work, Queensland University of Technology, Brisbane, Australia; 30 Department of Public Health, Universidad de los Andes, Santiago, Chile; 31 Centro Interdisciplinario de Cambio Global, Pontificia, Universidad Católica de Chile, Santiago, Chile; 32 Department of Environmental Health, School of Public Health, Fudan University, Shanghai, China; 33 Department of Environmental Health, University of São Paulo, São Paulo, Brazil; 34 Department of Primary Care and Population Health, University of Nicosia Medical School, Nicosia, Cyprus; 35 Institute of Atmospheric Physics, Academy of Sciences of the Czech Republic, Prague, Czech Republic; 36 Department of Family Medicine and Public Health, University of Tartu, Tartu, Estonia; 37 Estonian Environmental Research Centre, Tallinn, Estonia; 38 Santé Publique France, Department of Environmental Health, French National Public Health Agency, Saint Maurice, France; 39 Institute of Epidemiology, Helmholtz Zentrum München German Research Center for Environmental Health (GmbH), Neuherberg, Germany; 40 Department of Hygiene, Epidemiology and Medical Statistics, National and Kapodistrian University of Athens, Athens, Greece; 41 School of Public Health and Community Medicine, University of Gothenburg, Gothenburg, Sweden; 42 Geography and Urban Planning Department, University of Mazandaran, Babolsar, Iran; 43 Technological University Dublin, Dublin, Ireland; 44 UK Health Security Agency, London, UK; 45 Braun School of Public Health and Community Medicine, The Hebrew University of Jerusalem, Jerusalem, Israel; 46 Department of Epidemiology, Lazio Regional Health Service, Rome, Italy; 47 Department of Global Health Policy, Graduate School of Medicine, The University of Tokyo, Tokyo, Japan; 48 Department of Global Environmental Health, Graduate School of Medicine, University of Tokyo, Tokyo, Japan; 49 School of Tropical Medicine and Global Health, Nagasaki University, Nagasaki, Japan; 50 Center for Climate Change Adaptation, National Institute for Environmental Studies, Tsukuba, Japan; 51 Department of Environmental Health, Harvard T.H. Chan School of Public Health, Boston, MA USA; 52 Department of Environmental Health, National Institute of Public Health, Cuernavaca, Morelos, Mexico; 53 National Agency for Public Health of the Ministry of Health, Labour and Social Protection of the Republic of Moldova, Orhei Rayon, Republic of Moldova; 54 National Institute for Public Health and the Environment (RIVM), Centre for Sustainability and Environmental Health, Bilthoven, Netherlands; 55 Norwegian institute of Public Health, Oslo, Norway; 56 Institute of Tropical Medicine “Alexander von Humboldt”, Universidad Peruana Cayetano Heredia, Lima, Peru; 57 Department of Hygiene, Graduate School of Medicine, Hokkaido University, Sapporo, Japan; 58 Faculty of Geography, Babes-Bolay University, Cluj-Napoca, Romania; 59 Department of Earth Sciences, University of Torino, Torino, Italy; 60 Barcelona Institute for Global Health, Barcelona, Spain; 61 Department of Environmental Health. Rollins School of Public Health, Emory University, Atlanta, USA; 62 Department of Geography, Geoinformatics and Meteorology, University of Pretoria, Pretoria, South Africa; 63 Graduate School of Public Health, Seoul National University, Seoul, South Korea; 64 School of Biomedical Convergence Engineering, College of Information and Biomedical Engineering, Pusan National University, Yangsan, South Korea; 65 Institute of Environmental Assessment and Water Research (IDAEA), Spanish Council for Scientific Research (CSIC), Barcelona, Spain; 66 Department of Statistics and Computational Research. Universitat de València, València, Spain; 67 Department of Public Health and Clinical Medicine, Umeå University, Umeå, Sweden; 68 Swiss Tropical and Public Health Institute, Basel, Switzerland; 69 Department of Statistics, Computer Science and Applications “G. Parenti”, University of Florence, Florence, Italy; 70 Department of Quantitative Methods, School of Medicine, University of the Republic, Montevideo, Uruguay; 71 School of the Environment, Yale University, New Haven CT, USA; 72 Institute of Research and Development, Duy Tan University, Da Nang, Vietnam; 73 Department of Environmetal Health, Faculty of Public Health, University of Medicine and Pharmacy at Ho Chi Minh City, Ho Chi Minh City, Vietnam

**Keywords:** Environmental health, Projection and prediction, Environmental health

Correction to: *Nature Communications* 10.1038/s41467-023-40599-x, published online 24 August 2023

The original version of this Article omitted from the author list the 17th author, “Multi-Country Multi-City (MCC) collaborative research network”, which is the consortium providing the mortality data. A list of consortium authors and their affiliations are provided in the HTML version of this Correction. Part of the Author Contributions statement was incorrectly given and should have read ‘A.M.V.C., E.M.F., B.A., M.D.S.Z.S.C., Y.L.G., Y.G., Y.H., V.H., J.K., E.L., D.R., N.R., N.S., S.S., A.U., A.G. and the MCC were involved in resources and data curation.’

In addition, the primary affiliation ‘Climate Research Foundation (FIC), Madrid, Spain’ for Dominic Roye was missing.

