# Supplementary material for: Rapid increase in the risk of heat-related mortality
Source: Nat Commun. 2023 Aug 24;14:4894. doi: 10.1038/s41467-023-40599-x (PMC10449849; doi:10.1038/s41467-023-40599-x)
Supplement: Supplementary file 1 — Supplementary Information [file 41467_2023_40599_MOESM1_ESM.pdf]

Supplementary Information for article

Rapid increase in the risk of heat-related mortality

Samuel Lühi[1,2,*], Christopher Fairless[1], Erich M. Fischer3, Noah Scovronick[4] , Ben Armstrong[5], Micheline S. Z. S. Coelho[6], Yue Leon Guo[7,8,9], Yuming Guo[10], Yasushi Honda[11], Veronika Huber[12,13], Jan Kyselý[14,15], Eric Lavigne[16,17], Dominic RoyeÅL[18], Niilo Ryti[19], Susana Silva[20], Aleš Urban[14,15], Antonio Gasparrini[5,21,22], David N. Bresch[1,2], and Ana M. Vicedo-Cabrera[23,24,*]

[1]Institute for Environmental Decisions, ETH Zurich, Zurich, Switzerland
[2]Federal Office of Meteorology and Climatology, MeteoSwiss, Zurich-Airport, Switzerland
[3]Institute for Atmospheric and Climate Science, ETH Zurich, Zurich, Switzerland
[4]Gangarosa Department of Environmental Health. Rollins School of Public Health, Emory University, Atlanta, USA
[5]Department of Public Health Environments and Society, London School of Hygiene & Tropical Medicine, London, United Kingdom
[6]Department of Pathology, Faculty of Medicine, University of Saõ Paulo, Saõ Paulo, Brazil
[7]Environmental and Occupational Medicine, National Taiwan University (NTU) College of Medicine and NTU Hospital, Taipei, Taiwan
[8]National Institute of Environmental Health Science, National Health Research Institutes, Zhunan, Taiwan
[9]Graduate Institute of Environmental and Occupational Health Sciences, NTU College of Public Health, Taipei, Taiwan
[10]Climate, Air Quality Research Unit, School of Public Health and Preventive Medicine, Monash University, Melbourne, Australia
[11]Center for Climate Change Adaptation, National Institute for Environmental Studies, Tsukuba, Japan
[12]IBE-Chair of Epidemiology, LMU Munich, Munich, Germany
[13]Department of Physical, Chemical and Natural Systems, Universidad Pablo de Olavide, Sevilla, Spain
[14]Institute of Atmospheric Physics, Czech Academy of Sciences, Prague, Czech Republic
[15]Faculty of Environmental Sciences, Czech University of Life Sciences, Prague, Czech Republic
[16]School of Epidemiology & Public Health, Faculty of Medicine, University of Ottawa, Ottawa, Canada
[17]Environmental Health Science and Research Bureau, Health Canada, Ottawa, Canada
[18]Department of Geography, University of Santiago de Compostela, CIBER of Epidemiology and Public Health (CIBERESP), Spain
[19]Center for Environmental and Respiratory Health Research (CERH), University of Oulu, Oulu, Finland
[20]Department of Epidemiology, Instituto Nacional de SauÅLde Dr. Ricardo Jorge, Lisbon, Portugal
[21]Centre for Statistical Methodology, London School of Hygiene & Tropical Medicine, London, United Kingdom
[22]Centre on Climate Change & Planetary Health, London School of Hygiene & Tropical Medicine, London, United Kingdom
[23]Institute of Social and Preventive Medicine, University of Bern, Bern, Switzerland
[24]Oeschger Center for Climate Change Research, University of Bern, Bern, Switzerland

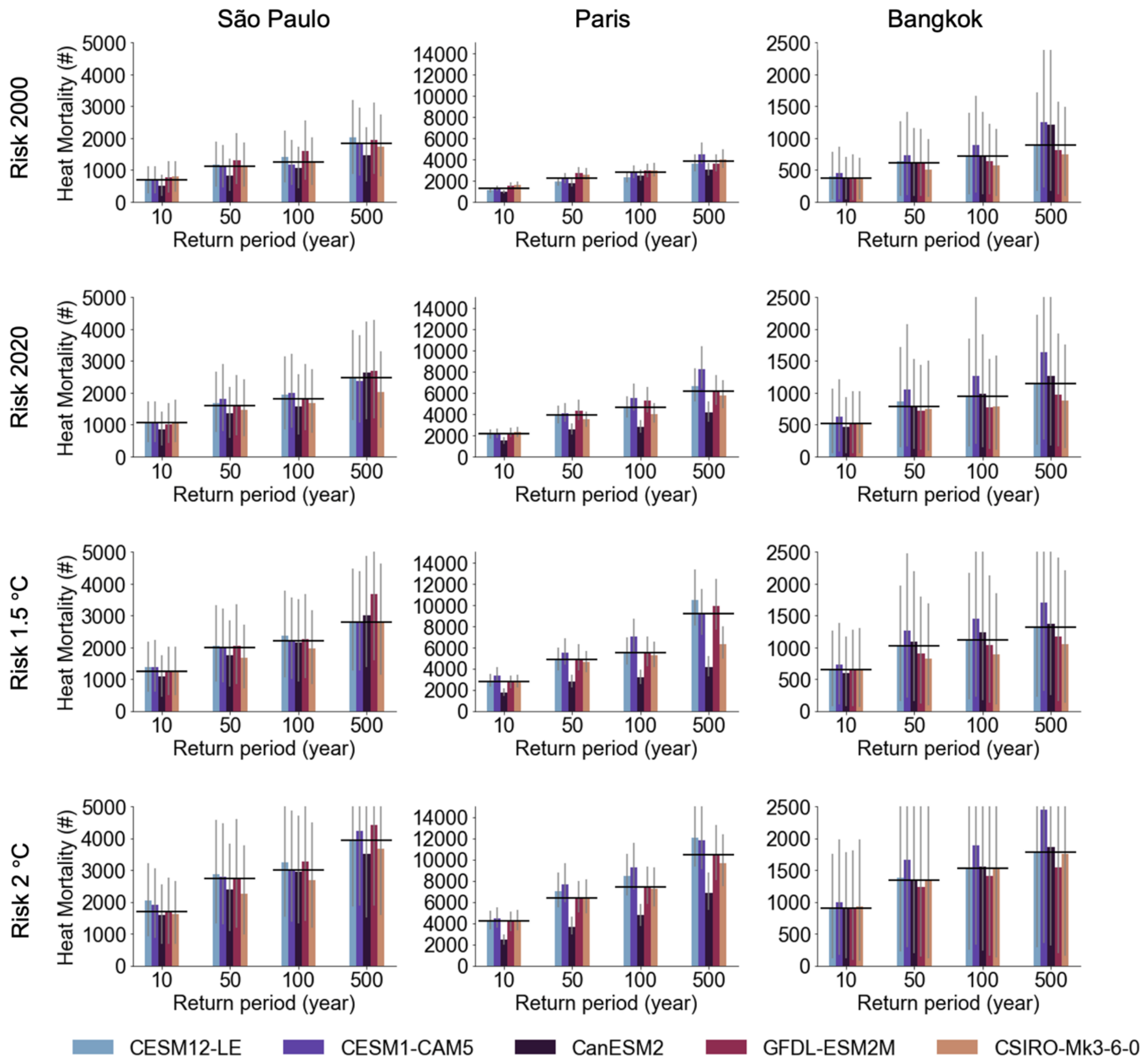

Figure S1: Heat mortality and uncertainty for different return periods for São Paulo (Brasil, left column), Paris (France, middle) and Bangkok (Thailand, right) and for warming levels of 2000 (0.7°C, top row), 2020 (1.2°C, second row), 1.5°C (third row) and 2.0°C (bottom row). Results are displayed for each SMILE (bars) including uncertainty estimates from the 95% confidence intervals from the relative risk associations (grey ticks).

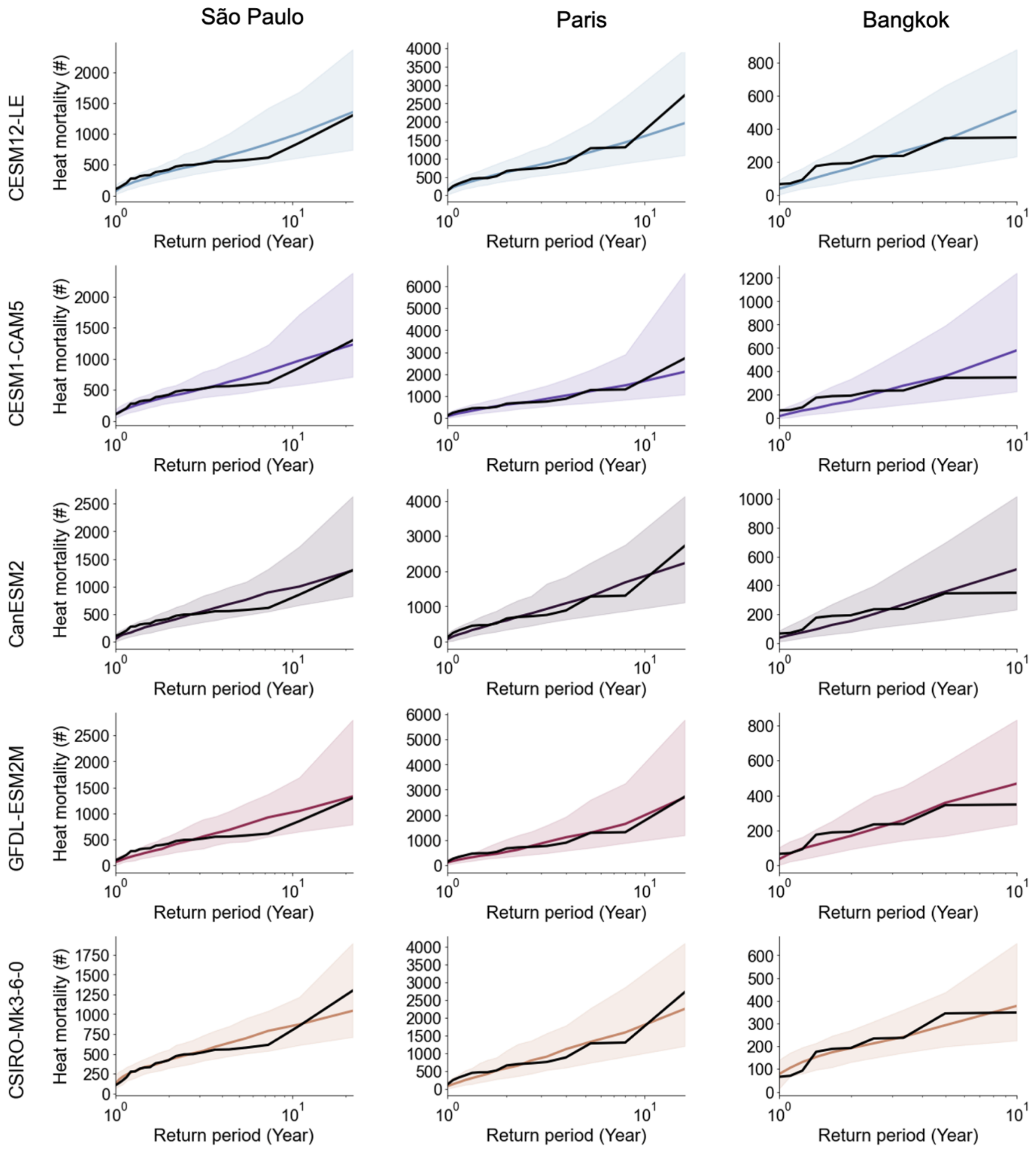

Figure S2: Stochastic uncertainty during the observational period within the impact exceedance frequency curves for São Paulo (Brasil, left column), Paris (France, middle) and Bangkok (Thailand, right) and all single-model initial-condition large ensembles (rows) used in this study. The shaded area is calculated by bootstrapping ensemble members and thus represents the 95% confidence interval from the uncertainty due to internal climate variability. All models capture the observational exceedance frequency curves well within their uncertainty interval.

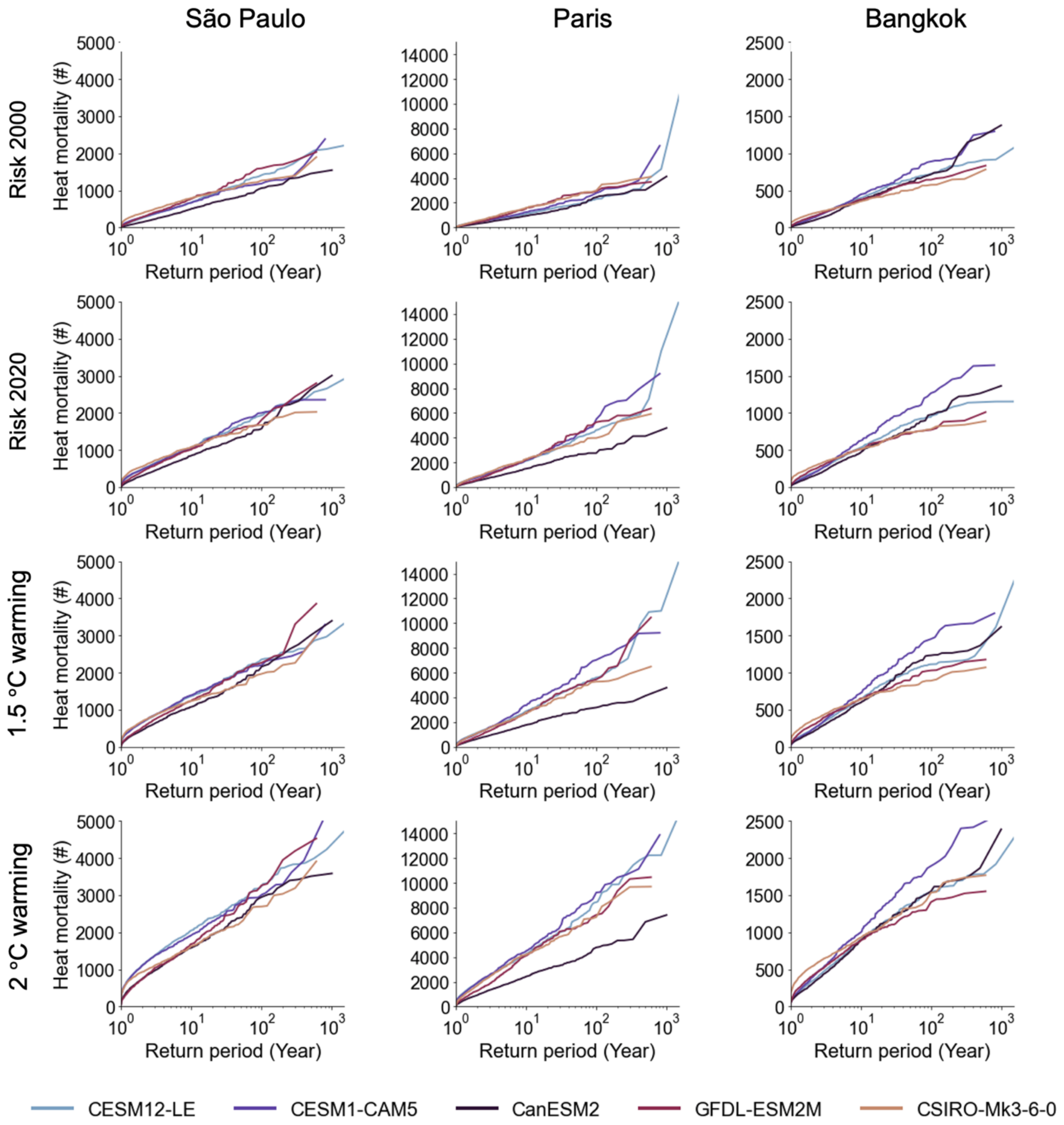

Figure S3: Impact exceedance frequency curves for São Paulo (Brasil, left column), Paris (France, middle) and Bangkok (Thailand, right) and for warming levels of 2000 (0.7°C, top row), 2020 (1.2◦C, second row), 1.5°C (third row) and 2.0°C (bottom row). Results are displayed for all five single-model initial-condition large ensembles used in this study.

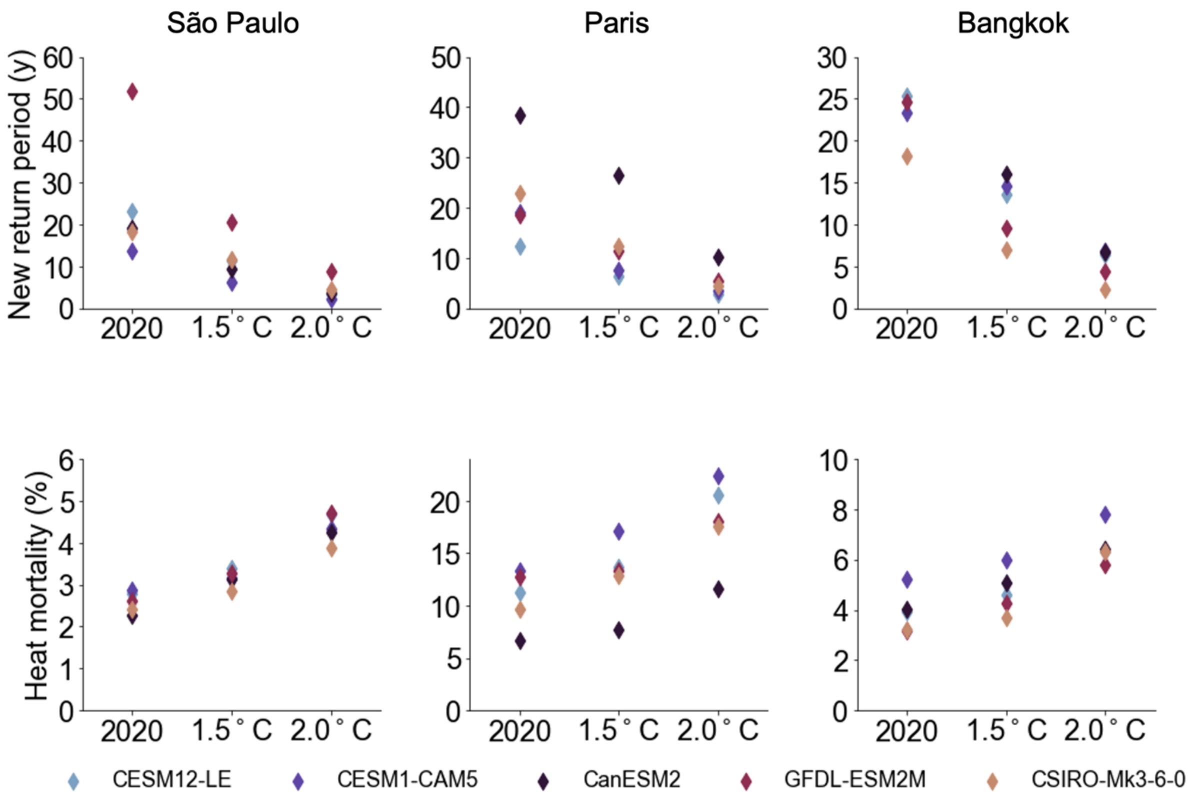

Figure S4: Changes of return periods for different warming levels compared to the risk in the climate of 2000 (top row) and share of heat mortality as percentage of overall mortality of a 1-in-100 year season (bottom row) for S.o Paulo (Brasil, left column), Paris (France, middle) and Bangkok (Thailand, right). Results are displayed for all five single-model initial-condition large ensembles used in this study.
