# Supplementary material for: Rapid increase in the risk of heat-related mortality
Source: Nat Commun. 2023 Aug 24;14:4894. doi: 10.1038/s41467-023-40599-x (PMC10449849; doi:10.1038/s41467-023-40599-x)
Supplement: Supplementary file 3 — Description of Additional Supplementary Files [file 41467_2023_40599_MOESM3_ESM.pdf]

**Description of Additional Supplementary Files**

File Name: Supplementary Data 1.

Description: Location-specific changes in return periods for different levels of warming, expressed as the new return period of the location specific 1-in-100 year heat-mortality level of 2000, and heat-mortality fraction of a 1-in-100 year season, expressed as fraction (%).
